# Association between body roundness index and strength fitness in senior middle school students

**DOI:** 10.3389/fpubh.2026.1669675

**Published:** 2026-02-10

**Authors:** Long Chen, Yiqun Yu, Chaoliang Mao, Jiali Xu, Zhixiu He, Hao Luo, Juncheng Zhu

**Affiliations:** 1 College of Physical Education and Health, East China Normal University, Shanghai, China; 2 School of Physical Education, Shangrao Normal University, Shangrao, China; 3 School of Teacher Education, Shangrao Normal University, Shangrao, China

**Keywords:** body roundness index, correlation research, regression analysis, strength quality, students

## Abstract

**Background:**

Adolescent obesity poses a major challenge to declining strength fitness. Body Roundness Index (BRI) provides superior quantification of central adiposity over traditional metrics. However, evidence regarding the BRI-strength fitness correlation remains underexplored in youth populations.Therefore, this study aims to explore the association between BRI levels and muscular fitness in senior middle school students.

**Methods:**

A cross-sectional study was conducted in 2024 among 3,822 senior senior middle school students (grades 10–12) from Jiangxi, Zhejiang, and Fujian provinces in China using stratified random cluster sampling. Data were collected through sociodemographic questionnaires, anthropometric measurements (height/waist circumference), and strength fitness tests (grip strength/sit-ups/standing long jump). Multivariable logistic regression and restricted cubic spline (RCS) models were used to analyze associations.

**Results:**

A total of 3,822 senior middle school students were included. Significant gender differences were found in grip strength, sit-ups, standing long jump, and BRI (all *P* < 0.01). The rates of substandard performance were 13.8% for grip strength, 19.7% for sit-ups, 17.4% for standing long jump, and 74.9% for overall strength fitness (*P*_75_ cutoff). Strength fitness deficits varied significantly across physical activity levels, grade, parental education, household income, and other sociodemographic factors (*P* < 0.05). Higher BRI levels (Q4) were associated with increased risk of failing sit-ups and standing long jump, especially in boys. BRI showed a non-linear dose–response relationship with grip strength, standing long jump, and overall strength fitness, and a linear negative association with sit-ups.

**Conclusion:**

An appropriate BRI level may contribute to the enhancement of strength-related fitness, while an excessively high BRI could potentially inhibit strength performance. Therefore, more attention should be payed on abdominal fat management and healthy dietary habits promotion and physical activity. Meanwhile, gender differences require personalized intervention strategies.

## 1 Introduction

In recent years, with the transformation of modern lifestyles, the issue of adolescent obesity has become increasingly severe, emerging as a critical public health challenge that demands urgent attention (1). The survey shows that the overweight and obesity rate among children and adolescents in China has risen from 0.1% in 1985 to 23.4% in 2019 (2). Meanwhile, relevant monitoring data indicates a significant decline in the strength fitness levels of adolescent students, specifically manifested by consecutive years of deteriorating test scores in standing long jump, pull-ups, and other assessments (3). Overweight and obesity are not only associated with metabolic disease risks (4), but studies also indicate that individuals with high BMI generally exhibit lower strength performance compared to their normal-weight peers due to elevated body fat percentage and restricted muscle function (5).

Although BMI is widely used to assess individual obesity levels, it does not account for fat distribution or specific body shape differences (6, 7). This limitation can obscure metabolic risks in individuals who have normal BMI but excessive body fat, and BMI fails to adequately reflect central (abdominal) obesity characteristics. The Body Roundness Index (BRI), which integrates waist circumference and height, provides a more precise quantification of trunk fat distribution (8). BRI has gained traction as an indicator of central adiposity in adult populations and has shown strong utility in chronic disease prediction; for instance, it exhibits a robust ability to predict metabolic syndrome and cardiovascular risk, outperforming BMI in some analyses (9). Consistent with this, measures of central obesity often correlate more strongly with chronic disease risk than BMI. Existing studies have confirmed that waist circumference and related indices of central obesity are better indicators of obesity-related health risks compared to BMI.

However, most prior research examining obesity and physical fitness in youth has relied predominantly on general adiposity indices such as BMI (10). Consequently, there is substantial evidence that higher adiposity is associated with poorer strength performance and reduced physical fitness in children and adolescents (11). In contrast, studies investigating the relationship between central obesity indicators like BRI and muscular fitness in the pediatric and adolescent population are still limited. Moreover, the specific mechanisms by which central obesity might affect muscle function remain unclear. This study aims to explore the association between BRI levels and muscular fitness in senior middle school students, thereby providing data support and a theoretical foundation for improving the physical health and strength fitness of adolescent students.

## 2 Subjects and methods

### 2.1 Subjects

From October to December 2024, a stratified random cluster sampling method was employed to conduct survey tests among students (grades 10–12) from 27 senior middle schools across three provinces: Jiangxi, Zhejiang, and Fujian. Nine senior middle schools were selected from each province. The inclusion criteria for the sample were: Registered students from the 27 senior middle schools in the three provinces; Randomly selected students from target classes in grades 10–12; Age conforming to the standard enrollment criteria for the corresponding grades (14–18 years old); Signed informed consent forms indicating voluntary participation. The exclusion criteria were: First, schools with a sample size of ≤ 5 (*n* = 22). Second, questionnaires missing 20% or more of key information (*n* = 54). Third, samples with response times of less than 150 s (*n* = 67). Fourth, samples exhibiting patterned responses across 10 or more consecutive items (*n* = 24) and finally, data presenting logical inconsistencies (*n* = 11). The surveyed schools covered the high school section, with one class randomly selected from each grade, each class comprising no fewer than 50 students. If the number was insufficient, students from adjacent classes of equivalent quality were added to meet the requirement. A total of 4,000 questionnaires were distributed. After systematic logical validation and manual double-checking, 178 invalid questionnaires were excluded, resulting in 3,822 valid questionnaires, yielding an effective response rate of 95.5%. Among them, there were 1,494 students (39.08%) in grade 10, 1,188 (31.08%) in grade 11, and 1,140 (29.82%) in grade 12; 2,005 (52.45%) were boarding students, while 1,817 (47.54%)were day students; 411 (10.75%) were only children, and 3,411(89.24%) were non-only children. All students participated voluntarily, having uniformly signed informed consent forms before the survey, with parental consent obtained. This study was approved by the Ethics Committee of Gannan Medical University (Approval No.: 2024724).

### 2.2 Method

#### 2.2.1 Basic information

The questionnaire content primarily includes basic information such as grade level, type of enrollment, registered residence location, whether an only child, parental education level, annual household income, physical activity level, and living conditions.

#### 2.2.2 Physical examination

The main measurements include height and waist circumference. When measuring height, the subject removes shoes and hats and stands upright on the stadiometer (Lejia Likang, model: HW-700E), with heels, the area between the shoulder blades, and the sacrum pressed firmly against the stadiometer's vertical column. The reading is taken when the pressure plate is level with the eyes. For waist circumference measurement, the subject stands erect with the abdomen relaxed, and the measuring tape is placed directly on the skin without clothing or a single layer of undergarment in between. The reading is taken at the end of each exhalation and recorded to the nearest 0.1 cm, with a permissible error of less than 0.5 cm between two measurements. After obtaining the accurate values for height and waist circumference, the BRI index is calculated using the BRI formula as follows: $BRI=364.2-365.5\times \sqrt{1-{\left(\frac{Waist\;Size\left(CM\right)}{2\times \Pi }\right)}^{2}}÷\sqrt{{\left(0.5\times Height\;\left(CM\right)\right)}^{2}}$ (12). The quartile method was employed for division, categorizing the body roundness index into four level intervals from Q1 to Q4, corresponding to progressively increasing degrees of body roundness from low to high.

#### 2.2.3 Physical strength quality

Strength quality refers to the ability of the body or a specific part of the body's muscles to overcome internal and external resistance during work (contraction and relaxation) (13). This study focuses on the upper limb, core abdominal, and lower limb muscle strength of individuals, assessing their overall comprehensive strength quality through three test items: grip strength, sit-ups, and standing long jump. For the grip strength test, a WCS-100 electronic dynamometer was used, with measurements taken on the dominant hand. Participants were instructed to stand naturally with legs slightly apart, arms hanging freely without touching clothing, and to exert steady force, with the stable reading from the dynamometer recorded. Three consecutive measurements were taken, and the best result was selected. During the sit-up test, participants lay supine with knees bent at 90°, hands crossed behind the head, while a partner pressed down on their ankles to stabilize the lower limbs. The test began and ended with a whistle signal, and the number of sit-ups completed within 30 s was recorded. The standing long jump test was conducted using a standing long jump tester (Hengkang:HK-6,000-TY). Participants stood behind the take-off line and jumped simultaneously with both feet, ensuring no toe-stepping on the line, hopping, or stepping during the process. Three consecutive attempts were made, with the best result selected. Measurement results for grip strength, sit-ups, and standing long jump were recorded with precision to 0.1 kg, 1 count, and 1 cm, respectively. Compliance with standards was determined based on the “New Evaluation Standards for Physical Health of Chinese Children and Adolescents (14).” Specific criteria were as follows: For boys aged 15–18, grip strength thresholds were ≥25.6 kg, ≥28.1 kg, ≥29.7 kg, and ≥30.9 kg, respectively; sit-ups required ≥18 counts; standing long jump thresholds were ≥176.2 cm, ≥183.2 cm, ≥186.3 cm, and ≥186.7 cm, respectively. For girls, grip strength thresholds were ≥17.9 kg, ≥18.4 kg, ≥19.0 kg, and ≥19.6 kg, respectively; sit-ups required ≥15 counts; standing long jump thresholds were ≥142.9 cm, ≥144.0 cm, ≥144.1 cm, and ≥143.6 cm, respectively. Failure to meet these standards was considered non-compliant (14). The strength quality index was calculated as: Z-grip strength +Z-sit-ups +Z-standing long jump. A positive index value indicated relatively better strength quality, while a negative value indicated relatively poorer performance. Comprehensive strength quality was evaluated by computing the strength quality index (15).

#### 2.2.4 Sleep disorder status and physical activity level

The Pittsburgh Sleep Quality Index (PSQI) (16) was used to assess the sleep quality of adolescents. This scale consists of 7 dimensions, with each dimension scored on a 0–3 scale, where different responses correspond to specific scores. A score of 0 indicates no problem or good condition, while a score of 3 indicates a severe problem. For example, in the sleep quality dimension, responding “slept very well” may yield a score of 0, whereas responding “slept very poorly” may result in a score of 3. The scores from all dimensions are summed to obtain a total score. A total score greater than 5 indicates the presence of sleep disturbances in adolescents. The PSQI demonstrates good reliability and validity, with a Cronbach's α coefficient of 0.87. For the assessment of participants' physical activity, the International Physical Activity Questionnaire Short-form (IPAQ-SF) (17) was employed. The metabolic equivalent (MET) value for walking is typically set at 3.3 METs, moderate-intensity activity at 4.0 METs, and vigorous-intensity activity at approximately 8.0 METs. The total weekly physical activity was calculated using the following formulas: Walking MET-min/week = walking days × walking duration (min) × 3.3; Moderate-intensity MET-min/week = moderate-intensity activity days × activity duration (min) × 4.0; Vigorous-intensity MET-min/week = vigorous-intensity activity days × activity duration (min) × 8.0. The MET-min/week values for these three activity intensities were then summed to derive the total physical activity volume. Based on the calculated total physical activity, participants were categorized into low, moderate, or high physical activity levels. The Cronbach's α coefficient for this questionnaire was 0.78.

#### 2.2.5 Beverage breakfast situation

A self-designed questionnaire was used to assess the breakfast frequency of adolescent students in the past week based on self-evaluation. The evaluation criteria were as follows: a weekly breakfast frequency of ≥4 times was classified as frequent breakfast consumption, while <4 times was classified as infrequent breakfast consumption. Additionally, referencing the “General Standard for Beverages” (18), a questionnaire was developed to investigate the beverage consumption frequency of respondents in the past week. The evaluation criteria were as follows: a weekly beverage consumption frequency of ≥4 times was classified as frequent beverage consumption, while <4 times was classified as infrequent beverage consumption.

### 2.3 Quality control

This survey was conducted electronically, administered collectively during students‘ free time after class to ensure convenient and efficient participation. Given the broad scope of the questionnaire, the completion time was set at 15–20 min to encourage careful responses. On-site staff were arranged to promptly address students' questions and issues, ensuring smooth completion. For data quality control, staff monitored response times through the questionnaire platform's backend, automatically flagging submissions under 5 min. After verification by quality inspectors, invalid responses were removed to ensure data reliability.

### 2.4 Statistical analysis

Survey data was exported using Questionnaire Star and analyzed with SPSS 27.0 software. Continuous numerical variables and categorical variables were described using (*M* ± *SD*), counts, and constituent ratios, respectively. *T*-tests, Mann-Whitney *U* tests, and χ^2^ tests were employed to compare the differences in basic characteristics of strength fitness and body roundness index among senior middle school students across groups. The χ^2^ test was used to compare the distribution of substandard rates of strength fitness across different demographic groups and to analyze the association between body roundness index levels and strength fitness in senior middle school students. Pearson and Spearman correlation analyses were conducted to examine the relationship between body roundness index and strength fitness. A binary logistic regression model was applied to explore the strength of association between body roundness index levels and strength fitness in senior middle school students. In binary logistic regression, we used 3 stepwise model. In model 3, physical activity level, frequency of breakfast consumption, presence of sleep disorders, and frequency of beverage consumption were added as confounding factors. Since these confounding factors have been reported to be associated with obesity and muscle strength (19–25). Additionally, R4.2.2 software was used to fit a restricted cubic spline model with quartiles as knots [3 knots( *P*_25_, *P*_50_, *P*_75_), Overall: 1.52, 2.01, 2.75; Male:1.52, 2.01, 2.75; Female: 1.50, 1.85, 2.37)] to analyze the dose-response relationship between body roundness index and strength fitness. The two-sided test significance level was set at α = 0.05.

## 3 Results

### 3.1 Basic characteristics of strength fitness and body roundness index among senior middle school students

There were statistically significant gender differences among senior middle school students in terms of grip strength, standing long jump, 30-s sit-ups, BRI, as well as whether grip strength, standing long jump, and sit-ups met the standards, strength quality index ≥*P*_75_, and BRI level groups(*t/*χ^2^values were 43.52, 54.07, 13.08, 6.49, 14.29, 10.30, 54.95, respectively; all *P-*values <0.01) see Table 1.

**Table 1:** Basic characteristics of strength quality and body roundness index of senior middle School Students.

**Group**	**Overall**	**Male**	**Female**	***t/Z/*χ^2^-value**	**Cohen's *d*/rank–biserial *r*/Cramér's *V* (95%*CI*)**	***P*-value**
Age^a^	16.23 ± 0.96	16.15 ± 0.95	16.21 ± 0.93	−1.81	−0.06 (-0.12~0.01)	0.070
Grip strength^a^	32.68 ± 12.05	38.99 ± 10.56	25.04 ± 8.92	**43.53**	**1.42 (1.34–1.49)**	**0.001**
Standing long jump^a^	189.14 ± 40.48	213.36 ± 34.96	159.81 ± 23.94	**54.08**	**1.76 (1.69–1.83)**	<**0.001**
30 S Sit–up^a^	21.72 ± 8.19	23.26 ± 8.20	19.85 ± 7.80	**13.08**	**0.43 (0.36–0.50)**	<**0.001**
Strength fitness index^b^	0.01(-1.22, 1.22)	0.07(-1.25, 1.30)	−0.12(-1.20, 1.17)	−1.59	−0.03 (-0.06–0.01)	0.111
BRI^a^	2.24 ± 1.22	2.36 ± 1.36	2.10 ± 1.00	**6.49**	**0.21 (0.15–0.26)**	<**0.001**
Whether grip strength meets the standard^c^				**14.29**	**0.06 (0.03–0.10)**	<**0.001**
Yes	3,294	1844 (88.1)	1450 (83.9)			
No	528	249 (11.9)	279 (16.1)			
Whether the standing long jump meets the standard^c^				20.37	0.07 (0.04–0.10)	<**0.001**
Yes	3,158	1782 (85.1)	1376 (79.6)			
No	664	311 (14.9)	353 (20.4)			
Whether the sit-up meet the standard^c^				0.03	0.00 (0.00–0.04)	0.853
Yes	3071	1684 (80.5)	1387 (80.2)			
No	751	409 (19.5)	342 (19.8)			
Whether the strength quality index≥$P_{75}^{\text{c}}$				**10.30**	**0.05 (0.02–0.09)**	**0.001**
Yes	959	568 (27.1)	391 (22.6)			
No	2863	1525 (72.9)	1338 (77.4)			
BRI Level/*n*^c^				**54.96**	**0.12 (0.09–0.16)**	<**0.001**
Q1	946	505 (24.1)	441 (25.5)			
Q2	961	466 (22.3)	495 (28.6)			
Q3	975	514 (24.6)	461 (26.7)			
Q4	940	608 (29.0)	332 (19.2)			

^a^Represents independent samples t-test. ^b^Represents Mann–Whitney U test, c represents χ^2^ chi-square test. Bold values indicate statistical significance (*p* < 0.05).

### 3.2 Comparison of the distribution of non-compliance rates in strength quality among senior middle school students across different demographic characteristic groups

Among 3,822 senior middle school students (grades 10–12), the detection rate of substandard grip strength was 13.81% (528/3,822), the detection rate of substandard sit-ups was 19.65% (748/3,822), the detection rate of substandard standing long jump was 17.37% (664/3,822), and the detection rate of strength quality index <P75 was 74.91% (2,863/3,822). There were statistically significant differences in strength quality substandard rates among different physical activity level groups (χ^2^ values = 37.97, 21.74, 13.10, 46.38, respectively) (all *P*-values <0.001). Statistically significant differences were observed in substandard sit-ups, standing long jump, and strength quality index <*P*_75_ among different grade levels, paternal education levels, and annual household income groups (χ^2^values = 14.20, 16.05, 24.62; 25.56, 8.25, 18.76; 32.026, 18.41, 56.49, respectively; all *P-*values <0.05). Statistically significant differences were found in substandard grip strength, sit-ups, and strength quality index <*P*_75_ among different household registration location groups (χ^2^values = 6.33, 16.87, 8.73; all *P-*values <0.05). Statistically significant differences were noted in substandard sit-ups and standing long jump among different school enrollment type groups (χ^2^values = 17.98, 15.24; all *P*-values <0.001). Statistically significant differences were observed in substandard sit-ups and strength quality index <P75 among different maternal education level groups (χ^2^values = 12.73, 10.69; all *P-*values <0.05). Additionally, there was a statistically significant difference in substandard standing long jump between groups based on frequent beverage consumption (χ^2^value = 4.12; *P*-value <0.05) see Table 2.

**Table 2:** Comparison of the distribution of non-compliance rates of senior middle School Students strength quality in different demographic characteristic groups.

**Group**	**Total number of people**	**Grip strength fails to meet the standard**	***x*^2^-value**	**Cramér's *V* (95%*CI*)**	***P-value***	**Sit-ups fail to meetthe standard**	***x*^2^-value**	**Cramér's *V* (95%*CI*)**	***P*-value**	**Standing long jump fails to meet the standard**	***x*^2^ -value**	**Cramér's *V* (95%*CI*)**	***P*-value**	**Strength quality index <*P*_75_**	***x*^2^ -value**	**Cramér's *V* (95%*CI*)**	***P*-value**
Grade			1.76	0.02 (0.01–0.06)	0.415		**14.20**	**0.06 (0.03–0.09)**	<**0.001**		**16.05**	**0.07 (0.04–0.10)**	<**0.001**		**24.63**	**0.08 (0.05–0.11)**	<**0.001**
Grade 10	1,494	220 (14.7)				312 (20.9)				271 (18.1)				1,162 (77.8)			
Grade 11	1,188	159 (13.4)				257 (21.6)				236 (19.9)				907 (76.3)			
Grade 12	1,140	149 (13.1)				182 (16.0)				157 (13.8)				794 (69.6)			
Type of enrollment			0.14	0.01 (0.00–0.04)	0.708		**17.99**	**0.07 (0.04–0.10)**	<**0.001**		**15.24**	**0.06 (0.03–0.09)**	<**0.001**		3.80	0.03 (0.00–0.06)	0.051
Boarding	2,005	273 (13.6)				446 (22.2)				394 (19.7)				1,528 (76.2)			
Day student	1,817	255 (14.0)				305 (16.8)				270 (14.9)				1,335 (73.5)			
Place of household registration			**6.33**	**0.04 (0.01–0.08)**	**0.012**		**16.87**	**0.07 (0.04–0.10)**	<**0.001**		2.12	0.02 (0.00–0.05)	0.145		**8.74**	**0.05 (0.01–0.08)**	**0.003**
Town	1,043	168 (16.1)				160 (15.3)				166 (15.9)				746 (71.5)			
Rural areas	2,779	360 (13.0)				591 (21.3)				498 (17.9)				2,117 (76.2)			
Is the person an only child			0.113	0.01 (0.00–0.04)	0.737		5.45	0.04 (0.01–0.07)	0.020		0.56	0.01 (0.00–0.04)	0.456		0.00	0.00 (0.00–0.04)	0.988
Yes	411	59 (14.4)				63 (15.3)				66 (16.1)				308 (74.9)			
No	3,411	469 (13.7)				688 (20.2)				598 (17.5)				2,555 (74.0.9)			
Father's educational background			3.28	0.03 (0.01–0.07)	0.351		**25.56**	**0.08 (0.06–0.11)**	<**0.001**		**8.26**	**0.05 (0.03–0.08)**	**0.041**		**18.77**	**0.07 (0.04–0.11)**	<**0.001**
Primary school and below	609	88 (14.4)				147 (24.1)				111 (18.2)				483 (79.3)			
Junior high school	2,190	302 (13.8)				451 (20.6)				388 (17.7)				1,651 (75.4)			
High school/secondary specialized school	724	89 (12.3)				117 (16.2)				131 (18.1)				530 (73.2)			
University and above	299	49 (16.4)				36 (12.0)				34 (11.4)				199 (66.6)			
Mother's educational background			7.34	0.04 (0.02–0.08)	0.062		**12.74**	**0.06 (0.04–0.09)**	**0.005**		5.04	0.04 (0.02–0.07)	0.169		**10.70**	**0.05 (0.03–0.09)**	**0.013**
Primary school and below	1,084	136 (12.5)				242 (22.3)				174 (16.1)				833 (76.8)			
Junior high school	1,921	257 (13.4)				375 (19.5)				345 (18.0)				1,451 (75.5)			
High school/Secondary specialized school	606	97 (16.0)				107 (17.7)				116 (19.1)				435 (71.8)			
University and above	211	38 (18.0)				27 (12.8)				29 (13.0.7)				144 (68.2)			
Annual household income			4.37	0.03 (0.02–0.07)	0.224		**32.06**	**0.09 (0.06–0.13)**	<**0.001**		**18.41**	**0.07 (0.04–0.10)**	<**0.001**		**56.50**	**0.12 (0.09–0.16)**	<**0.001**
≤ 50,000	1,900	282 (14.8)				434 (22.8)				373 (19.6)				1,514 (79.7)			
51,000–100,000	967	118 (12.2)				183 (18.9)				154 (15.9)				708 (73.2)			
101,000–300,000	824	108 (13.1)				113 (13.7)				110 (13.3)				558 (67.7)			
≥301,000	131	20 (15.3)				21 (16.0)				27 (20.6)				83 (63.4)			
Physical activity level			**37.98**	**0.10 (0.07–0.13)**	<**0.001**		**21.75**	**0.08 (0.05–0.11)**	<**0.001**		**13.10**	**0.06 (0.03–0.09)**	<**0.001**		**46.38**	**0.11 (0.08–0.14)**	<**0.001**
Low level	1,639	184 (11.2)				279 (17.0)				292 (17.8)				1,277 (77.9)			
Intermediate level	1,083	133 (12.3)				206 (19.0)				153 (14.1)				729 (67.3)			
High level	1,100	211 (19.2)				266 (24.2)				219 (19.9)				857 (77.9)			
Whether sleep disorder			0.14	0.01 (0.00–0.04)	0.710		0.05	0.00 (0.00–0.04)	0.831		0.55	0.01 (0.00–0.04)	0.458		1.64	0.02 (0.00–0.05)	0.201
Yes	268	35 (13.1)				54 (20.1)				51 (19.0)				192 (71.6)			
No	3,554	493 (13.9)				697 (19.6)				613 (17.2)				2,671 (75.2)			
Whether often eat breakfast			0.60	0.01 (0.00–0.04)	0.440		2.68	0.03 (0.00–0.6)	0.101		0.05	0.00 (0.00–0.04)	0.831		0.09	0.01 (0.00–0.04)	0.766
Yes	3,614	503 (13.9)				701 (1,934)				629 (17.4)				2,709 (75.0)			
No	208	25 (12.0)				50 (24.0)				35 (16.8)				154 (74.0)			
Whether often drink beverages			0.02	0.002 (0.000–0.036)	0.896		0.45	0.01 (0.00–0.04)	0.502		**4.12**	**0.03 (0.00–0.07)**	**0.042**		0.33	0.00 (0.00–0.04)	0.856
Yes	1,501	206 (13.7)				303 (20.2)				284 (18.9)				1,122 (74.8)			
No	2,321	322 (13.9)				448 (19.3)				380 (16.4)				1,741 (75.0)			

The content within parentheses indicates the detection rate. Bold values indicate statistical significance (*p* < 0.05).

### 3.3 The association between body roundness index levels and strength fitness in senior middle school students

There is an association between different BRI level groups and failure to meet standards in grip strength, sit-ups, and standing long jump among senior middle school students overall and male students (χ^2^values = 8.942, 32.766, 21.451; 8.637, 19.209, 17.797, respectively, all *P*-values <0.05). Specifically, when the BRI index of senior middle school students overall falls within the Q4 level range, the detection rates of individuals failing to meet standards in grip strength, sit-ups, and standing long jump are the highest within the same group see Table 3.

**Table 3:** The association between the body roundness index level and strength quality of senior middle School Students.

**Gender**	**Group**	**Total number of people**	**Grip strength fails to meet the standard**	***x*^2^-value**	**Cramér's *V* (95%*CI*)**	***P*-value**	**Sit-ups fail to meetthe standard**	***x*^2^-value**	**Cramér's *V* (95%*CI*)**	***P*-value**	**Standing long jump fails to meet the standard**	***x*^2^-value**	**Cramér's *V* (95%*CI*)**	***P*-value**	**Strength quality index <*P*_75_**	***x*^2^-value**	**Cramér's *V* (95%*CI*)**	***P*-value**
Male	BRI Level	2,093	249 (11.9)	**8.942**	**0.07 (0.03–0.11)**	**0.030**	409 (19.5)	**32.766**	**0.13 (0.09–0.17)**	<**0.001**	311 (14.9)	**21.451**	**0.10 (0.06–0.15)**	<**0.001**	1,525 (72.9)	2.504	0.04 (0.02–0.09)	0.474
	Q1	505	73 (14.5)				90 (17.8)				69 (13.7)				368 (72.9)			
	Q2	466	50 (10.7)				77 (16.5)				56 (12.0)				339 (72.7)			
	Q3	514	46 (8.9)				77 (15.0)				62 (12.1)				363 (70.6)			
	Q4	608	80 (13.2)				165 (27.1)				124 (20.4)				455 (74.8)			
Female	BRI Level	1,729	279 (16.1)	3.560	0.05 (0.02–0.10)	0.313	342 (19.8)	0.507	0.02 (0.01–0.08)	0.917	353 (20.4)	7.270	0.07 (0.03–0.12)	0.064	1,338 (77.4)	1.116	0.03 (0.01–0.08)	0.773
	Q1	441	78 (17.7)				84 (19.0)				82 (18.6)				338 (76.6)			
	Q2	495	69 (13.9)				103 (20.8)				113 (22.8)				388 (78.4)			
	Q3	461	72 (15.6)				90 (19.5)				80 (17.4)				351 (76.1)			
	Q4	332	60 (18.1)				65 (19.6)				78 (23.5)				261 (78.6)			
Overall	BRI Level			**8.637**	**0.05 (0.02–0.08)**	**0.035**		**19.209**	**0.07 (0.04–0.11)**	<**0.001**		**17.797**	**0.07 (0.04–0.10)**	<**0.001**		2.577	0.03 (0.01–0.06)	0.462
	Q1	946	151 (16.0)				174 (18.4)				151 (16.0)				706 (74.6)			
	Q2	961	119 (12.4)				180 (18.7)				169 (17.6)				727 (75.7)			
	Q3	975	118 (12.1)				167 (17.1)				142 (14.6)				714 (73.2)			
	Q4	940	140 (14.9)				230 (24.5)				202 (21.5)				716 (76.2)			

The content within parentheses indicates the detection rate. Bold values indicate statistical significance (*p* < 0.05).

### 3.4 Correlation analysis between body roundness index levels and strength fitness in senior middle school students

Correlation analysis showed that senior middle school students' BRI was positively correlated with grip strength (r = 0.077, *P*-values <0.01), but negatively correlated with sit-ups, standing long jump, and strength quality index (r = −0.051,−0.047,−0.033, all *P*-values <0.05) see Table 4.

**Table 4:** Analysis of the correlation between body roundness index and strength qualities in senior middle school students.

**Variable**	**BRI**	**Grip strength**	**Sit-up**	**Standing long jump**	**Strength quality index**
BRI^a^	1	–	–	–	–
Grip strength^a^	0.077^**^	1	–	–	–
Sit–up^a^	−0.051^**^	0.203^**^	1	–	–
Standing long jump^a^	−0.047^**^	0.515^**^	0.322^**^	1	–
Strength Quality Index^b^	−0.033^**^	0.501^**^	0.639^**^	0.506^**^	1

^a^Represents Pearson correlation analysis.

^b^Represents Spearman correlation analysis.

^**^P < 0.01.

^*^P < 0.05.

### 3.5 Binary logistic regression analysis of body roundness index levels and strength fitness in senior middle school students

Using the BRI level of senior middle school students (boys, girls, and overall) as the independent variable, logistic regression models were established with strength fitness (grip strength, sit-ups, standing long jump, and comprehensive strength fitness) meeting the standard [yes = 1 (reference), no = 2] as the dependent variable. The independent variable was assigned as follows: BRI level [(Models 1, 2, 3) Q1 = 1 (reference), Q2 = 2, Q3 = 3, Q4 = 4]. After adjusting for confounding factors, the results of Model 3 showed that compared with the reference in the same group, when the BRI level of senior middle school students was in the Q4 range, the risk of failing to meet the standards for sit-ups and standing long jump performance increased (overall, sit-ups: *OR* = 1.432, *95%CI*:1.142–1.795, standing long jump: *OR* = 1.527, *95%CI*:1.204–1.937, respectively; boys, sit-ups: *OR* = 1.762, *95%CI*:1.311–2.367, standing long jump: *OR* = 1.617, *95%CI*:1.168–2.238, respectively, all *P*-values <0.01). Compared with the body roundness index in the Q1 range, the risk of failing to meet the standard for grip strength performance was lower when the overall BRI was in the Q2 and Q3 ranges and boys' BRI was in the Q3 range (overall, Q3: *OR* = 0.750, *95%CI*:0.577-0.974, Q2: *OR* = 0.734, *95%CI*:0.564–0.954, respectively; boys, *OR* = 0.590, *95%CI*:0.397–0.876, all *P*-values <0.05). For girls, the association differences between various BRI levels and strength fitness meeting the standards were not statistically significant (all *P*-values >0.05) see Table 5.

**Table 5:** Binary logistic regression analysis of the body roundness index level and strength quality of senior middle school students.

**Gender**	**BRI Level**	**Grip strength fails to meet the standard**			**Failed to meet the sit–up standard**			**Standing long jump fails to meet the standard**			**Comprehensive physical strength fails to meet the standard**		
		β**–value**	***OR*** **–value (95%** ***CI*** **)**	***P–value***	β**–value**	***OR*** **–value (95%** ***CI*** **)**	***P*** **–value**	β**–value**	***OR*** **–value (95%** ***CI*** **)**	***P*** **–value**	β**–value**	***OR*** **–value (95%** ***CI*** **)**	***P*** **–value**
Male	Model 1												
	Q4	−0.109	0.897 (0.637–1.262)	0.532	0.541	1.717 (1.286–2.294)	<**0.001**	0.482	1.619 (1.174–2.233)	**0.003**	0.102	1.107 (0.847–1.448)	0.457
	Q3	−0.542	0.582 (0.393–0.860)	**0.007**	−0.208	0.812 (0.583–1.133)	0.221	−0.143	0.867 (0.600–1.251)	0.445	−0.111	0.895 (0.681–1.176)	0.426
	Q2	−0.341	0.711 (0.484–1.044)	0.082	−0.091	0.913 (0.653–1.275)	0.592	−0.147	0.863 (0.592–1.259)	0.444	−0.006	0.994 (0.749–1.319)	0.965
	Q1		1			1			1			1	
	Model 2												
	Q4	−0.099	0.906 (0.642–1.279)	0.574	**0.575**	1.776 (1.325–2.381)	<**0.001**	0.497	1.644 (1.189–2.274)	**0.003**	0.168	1.183 (0.899–1.557)	0.231
	Q3	−0.534	0.586 (0.395–0.870)	**0.008**	−0.200	0.818 (0.585–1.145)	0.242	−0.139	0.870 (0.601–1.259)	0.460	−0.069	0.934 (0.706–1.235)	0.631
	Q2	−0.318	0.728 (0.494–1.073)	0.108	−0.060	0.942 (0.672–1.321)	0.729	−0.149	0.862 (0.589–1.260)	0.443	0.052	1.053 (0.788–1.407)	0.727
	Q1		1			1			1			1	
	Model 3												
	Q4	−0.118	0.889 (0.629–1.256)	0.504	0.566	1.762 (1.311–2.367)	<**0.001**	0.481	1.617 (1.168–2.238)	**0.004**	0.179	1.196 (0.908–1.576)	0.204
	Q3	−0.528	0.590 (0.397–0.876)	**0.009**	−0.182	0.833 (0.595–1.168)	0.290	−0.129	0.879 (0.607–1.273)	0.495	−0.076	0.927 (0.701–1.226)	0.596
	Q2	−0.308	0.735 (0.498–1.084)	0.121	−0.034	0.966 (0.688–1.358)	0.843	−0.132	0.876 (0.598–1.282)	0.496	0.040	1.040 (0.778–1.391)	0.789
	Q1		1			1			1			1	
Female	Model 1												
	Q4	0.026	1.027 (0.708–1.488)	0.890	0.034	1.035 (0.721–1.484)	0.853	0.296	1.344 (0.948–1.906)	0.097	0.114	1.120 (0.795–1.578)	0.516
	Q3	−0.149	0.861 (0.607–1.223)	0.404	0.031	1.031 (0.741–1.435)	0.857	−0.084	0.919 (0.654–1.292)	0.628	−0.028	0.972 (0.715–1.322)	0.858
	Q2	−0.283	0.754 (0.530–1.073)	0.116	0.110	1.117 (0.809–1.541)	0.501	0.259	1.295 (0.942–1.781)	0.112	0.100	1.105 (0.813–1.503)	0.524
	Q1		1			1			1			1	
Model 2												
Q4	0.003	1.003 (0.689–1.461)	0.987	0.000	1.000 (0.692–1.445)	0.999	0.290	1.336 (0.936–1.907)	0.111	0.097	1.101 (0.777–1.562)	0.588
Q3	−0.157	0.855 (0.600–1.218)	0.385	−0.001	0.999 (0.714–1.399)	0.996	−0.077	0.925 (0.656–1.306)	0.660	−0.056	0.946 (0.692–1.292)	0.725
Q2	−0.288	0.750 (0.526–1.069)	0.112	0.095	1.100 (0.794–1.524)	0.567	0.241	1.273 (0.922–1.758)	0.143	0.073	1.076 (0.788–1.468)	0.646
Q1		1			1			1			1	
Model 3												
Q4	−0.035	0.965 (0.660–1.412)	0.855	−0.022	0.979 (0.676–1.418)	0.909	0.320	1.377 (0.961–1.972)	0.081	0.092	1.096 (0.772–1.557)	0.608
Q3	−0.135	0.874 (0.611–1.250)	0.460	0.011	1.011 (0.721–1.416)	0.951	−0.075	0.928 (0.656–1.313)	0.673	−0.044	0.957 (0.700–1.309)	0.785
Q2	−0.266	0.767 (0.536–1.096)	0.146	0.089	1.093 (0.788–1.516)	0.595	0.239	1.270 (0.917–1.758)	0.151	0.071	1.074 (0.786–1.467)	0.655
Q1		1			1			1			1	
Overall	Model 1												
Q4	−0.082	0.921 (0.718–1.183)	0.521	0.363	1.437 (1.151–1.794)	**0.001**	0.365	1.441 (1.141–1.820)	**0.002**	0.083	1.087 (0.881–1.340)	0.438
Q3	−0.322	0.725 (0.559–0.940)	**0.015**	−0.087	0.917 (0.726–1.159)	0.468	−0.108	0.897 (0.700–1.151)	0.394	−0.073	0.930 (0.758–1.140)	0.485
Q2	−0.296	0.744 (0.574–0.964)	**0.025**	0.022	1.023 (0.812–1.288)	0.850	0.116	1.123 (0.883–1.429)	0.343	0.055	1.056 (0.858–1.300)	0.606
Q1		1			1			1			1	
Model 2												
Q4	−0.045	0.956 (0.742–1.231)	0.727	0.378	1.459 (1.165–1.828)	<**0.001**	0.415	1.515 (1.195–1.920)	<**0.001**	0.119	1.127 (0.910–1.395)	0.273
Q3	−0.324	0.723 (0.557–0.939)	**0.015**	−0.097	0.908 (0.716–1.150)	0.421	−0.113	0.893 (0.695–1.149)	0.379	−0.072	0.930 (0.756–1.144)	0.494
Q2	−0.308	0.735 (0.566–0.954)	**0.021**	0.032	1.033 (0.818–1.304)	0.786	0.090	1.094 (0.858–1.395)	0.467	0.067	1.069 (0.866–1.320)	0.533
Q1		1			1			1			1	
Model 3												
Q4	−0.070	0.932 (0.723–1.202)	0.589	0.359	1.432 (1.142–1.795)	**0.002**	0.424	1.527 (1.204–1.937)	<**0.001**	0.123	1.131 (0.913–1.400)	0.261
Q3	−0.310	0.734 (0.564–0.954)	**0.021**	−0.088	0.916 (0.723–1.161)	0.468	−0.109	0.897 (0.697–1.153)	0.396	−0.075	0.928 (0.754–1.141)	0.478
Q2	−0.288	0.750 (0.577–0.974)	**0.031**	0.042	1.043 (0.825–1.318)	0.725	0.090	1.094 (0.858–1.396)	0.467	0.061	1.063 (0.861–1.312)	0.573
Q1		1			1			1			**1**	

Model 1 is the crude model without adjusted factors.

Model 2 adjusted for age, grade, household registration location, type of schooling, only-child status, father's education level, mother's education level, and total annual household income, with an additional adjustment for gender across the overall sample.

Model 3 adjusted for age, grade, household registration location, type of schooling, whether an only child, father's education level, mother's education level, annual household income, physical activity level, frequency of breakfast consumption, presence of sleep disorders, and frequency of beverage consumption, with an additional overall adjustment for gender. Bold values indicate statistical significance (*p* < 0.05).

### 3.6 The dose-response relationship between body roundness index and strength fitness in senior middle school students

**Figure 1:**
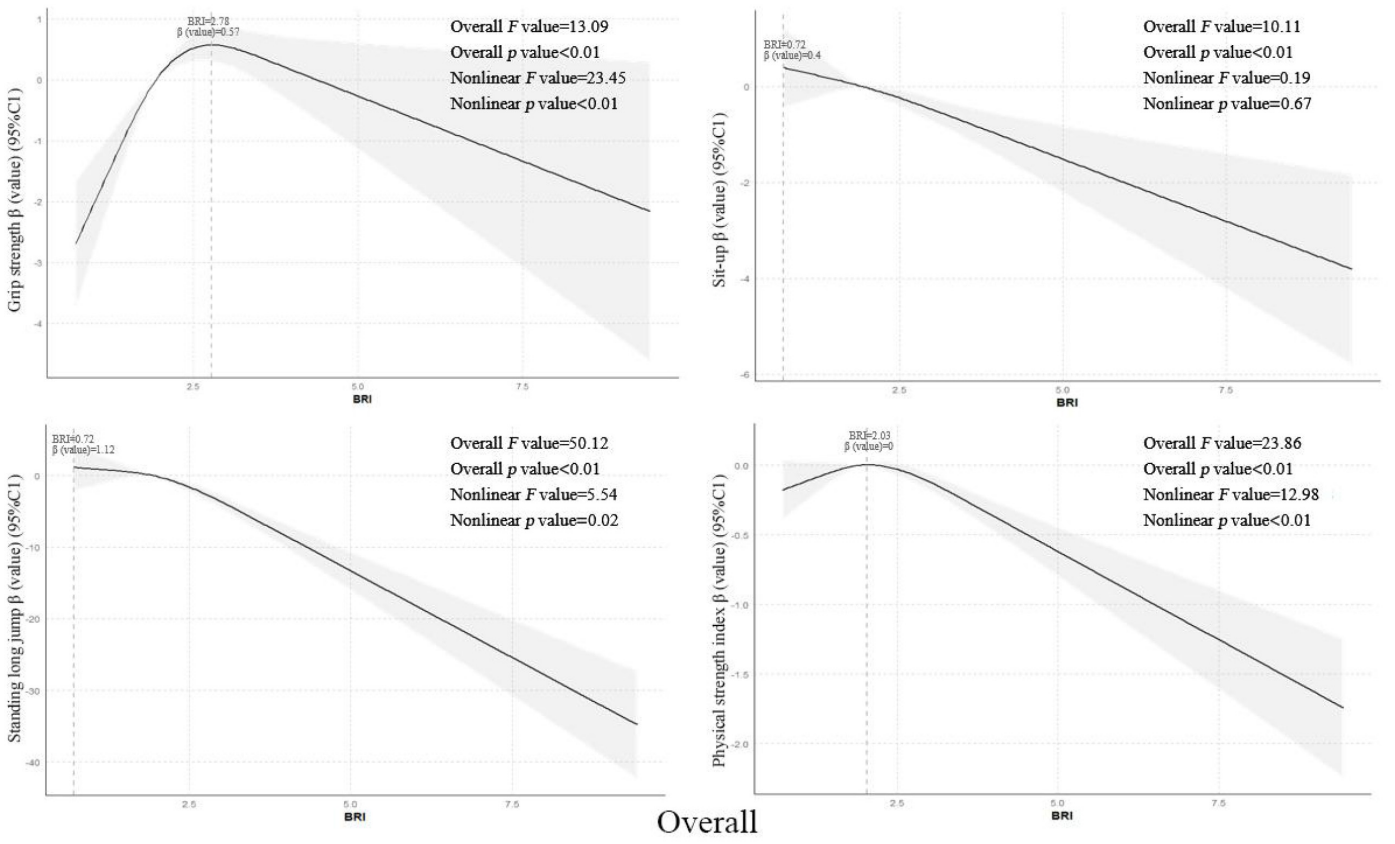
Dose-response relationship between Body Roundness Index and muscular fitness in senior middle school students (Overall).

**Figure 2:**
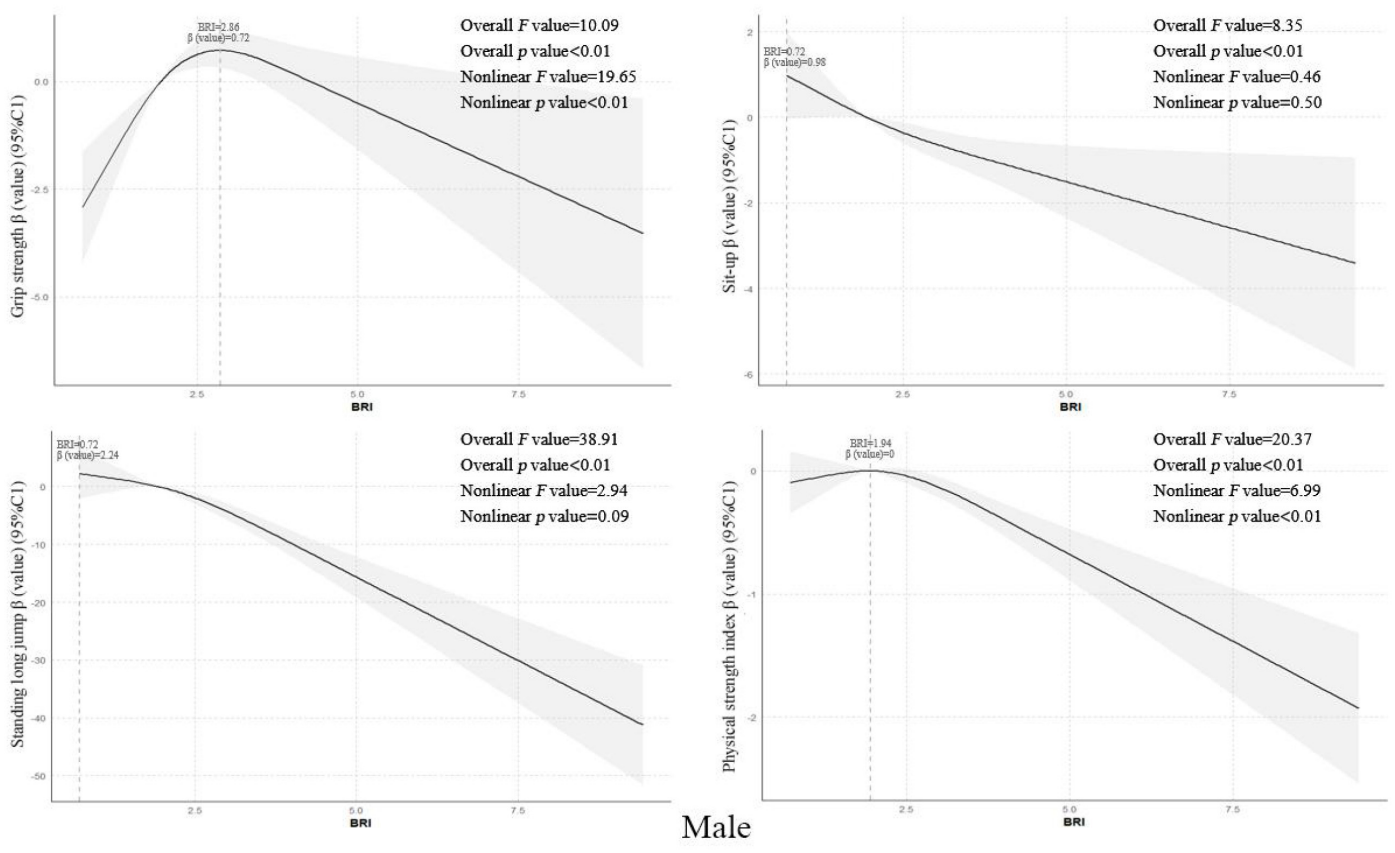
Dose-response relationship between Body Roundness Index and muscular fitness in senior middle school students (Boys).

**Figure 3:**
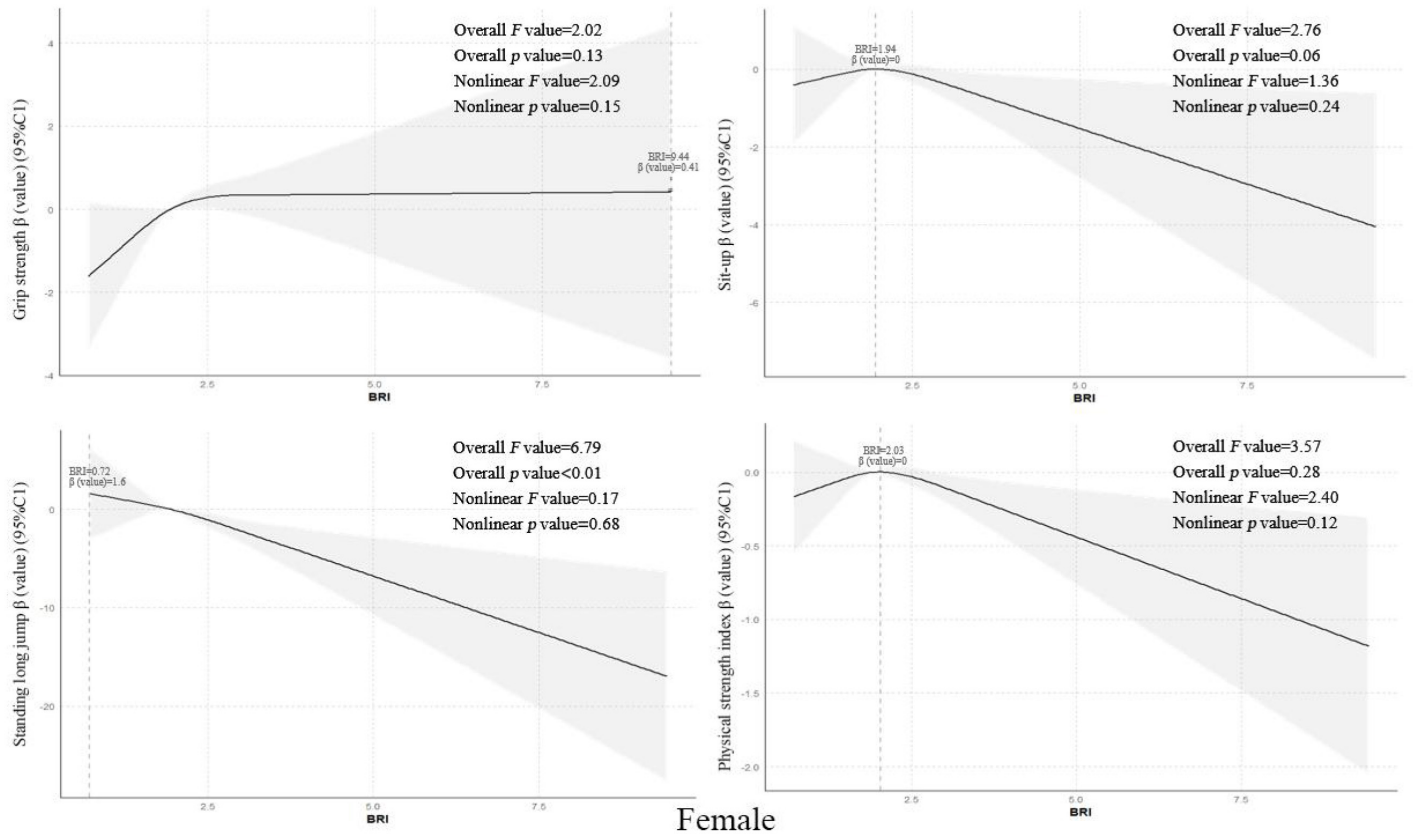
Dose-response relationship between Body Roundness Index and muscular fitness in senior middle school students (Girls).

Using restricted cubic spline models while controlling for covariates such as grade level, enrollment type, household registration location, only-child status, sleep disorders, regular breakfast consumption, frequent beverage intake, parental education level, annual household income, and physical activity level, the results showed that the overall BRI values of senior middle school students exhibited non-linear dose-response relationships with grip strength, standing long jump, and strength quality index (all *p*-trend values <0.01, all *p*-non-linear values <0.05), with optimal BRI values of 2.78, 0.72, and 2.03 respectively. However, a linear negative correlation was observed with sit-ups (*p*-trend value <0.01). Further gender stratification revealed that male students‘ BRI values showed non-linear dose-response relationships with grip strength and strength quality index (both *p*-trend values <0.01, both *p*-non-linear values <0.01), with optimal BRI values of 2.86 and 1.94 respectively, while linear negative correlations were found with standing long jump and sit-ups (*p*-trend value <0.01) see Figure 2. Female students' BRI values demonstrated a linear negative correlation with standing long jump (*p*-trend value <0.01) see Figures 1-3.

## 4 Discussion

In recent years, the physical health of adolescents has garnered increasing attention, particularly the decline in strength-related fitness, which has become a significant factor affecting their physical and mental development. Research indicates that as the Body Roundness Index (BRI) increases, adolescents exhibit unfavorable trends in strength-related fitness, especially in core strength (e.g., sit-ups) and lower limb strength (e.g., standing long jump). Therefore, schools and relevant departments should prioritize adolescents' BRI levels, regularly assess their strength-related fitness, and implement scientific physical exercise and dietary interventions to help them maintain appropriate BRI levels and promote the healthy development of their strength-related fitness.

This study reveals that senior middle school students exhibit relatively high failure rates in strength-related fitness tests such as grip strength, sit-ups, and standing long jump, with rates of 13.81% (528/3,822), 19.65% (748/3,822), and 17.37% (664/3,822), respectively, which align with findings from related studies (26). This indicates an urgent need to focus on improving the strength fitness of adolescents. Univariate analysis results demonstrate a significant correlation between physical activity levels and the failure rates in various strength fitness tests, with the low physical activity group showing higher failure rates, consistent with prior research (27). In addition, the detection rate of strength quality index <P75 was 74.91% (2,863/3,822), which was different from a previous domestic study that substandard rate of comprehensive strength <P_10_ was 10.0%(816/8 180) (28). The distinction is contributed to difference standard classification. Furthermore, urban-rural disparities, higher parental education levels, and favorable family economic conditions may provide adolescents with greater access to physical exercise resources and nutritional support, thereby influencing the development of strength fitness (29, 30). Logistic regression analysis further indicates that when senior middle school students' BRI levels fall within the Q4 range, the risk of failing sit-ups and standing long jump tests increases, which may be associated with excessive abdominal fat accumulation, further impairing strength performance. As a key indicator of abdominal fat deposition, BRI is positively correlated with body fat percentage (6). Excessive abdominal fat may adversely affect strength fitness through multiple mechanisms. Research suggests that abdominal fat accumulation secretes cytokines (e.g., TNF-α, IL-6), triggering inflammatory responses and inhibiting muscle protein synthesis, thereby reducing muscle strength (31). Furthermore, excessive body weight can impair muscle movement efficiency and interfere with the nervous system's control over muscles, limiting strength output (32). In strength tests involving body weight, such as sit-ups and standing long jump, the additional burden of abdominal fat becomes particularly pronounced, potentially further suppressing strength performance (33).

Although BMI is a traditional indicator for weight assessment, it has certain limitations in reflecting fat distribution. Compared with the traditional Body Mass Index (BMI), the Body Roundness Index (BRI) can more accurately characterize abdominal fat distribution (5). The results of this study show that the optimal BRI range (BRI = 2.03–2.78) is lower than the threshold identified in previous studies (4.62) (34). The difference of optimal BRI range may be caused by different subjects that our subjects were senior middle school students, while those of previous studies were adults. It suggests that different standards may apply for optimizing muscular strength and preventing chronic diseases. When BRI exceeds 2.78, although it does not reach the risk threshold for metabolic diseases, it may already begin to adversely affect the muscular strength of senior middle school students. The optimal BRI value in this study reflects a balance between fat reserves and energy metabolism. This phenomenon may stem from the fact that moderate fat reserves can serve as an energy source during strength training or high-intensity exercise, providing metabolic support for muscles and thereby enhancing strength performance (35, 36). However, excessively high BRI may lead to excessive abdominal fat accumulation, which could then inhibit muscle strength through mechanisms such as systemic inflammatory responses, muscle metabolic disorders, and impaired neural control (31). Maintaining BRI within a specific range (overall 2.03–2.78; males 1.94–2.86) is key to balancing fat metabolism and strength maintenance. Therefore, controlling BRI levels and avoiding excessive abdominal fat accumulation may be an important strategy for improving muscular strength in senior middle school students. Gender-stratified results indicate that males perform best at BRI values of 2.86 (grip strength) and 1.94 (strength index), while females only show a linear negative correlation between BRI and standing long jump. This difference may be related to distinct physiological characteristics between genders. Males have higher muscle mass, where moderate fat reserves can optimize energy metabolism, whereas hormonal fluctuations during female adolescence may weaken the direct association between BRI and strength, instead being regulated by muscle mass and hormone levels (37). Thus, health interventions for adolescents should incorporate gender-specific strategies, keeping BRI within an appropriate range (e.g., overall 2.03–2.78; males 1.94–2.86) while avoiding exceeding the threshold for visceral fat accumulation (e.g., ≥4.62) (34), thereby balancing both muscular strength enhancement and chronic disease prevention.

The strength of our study is that the association between BRI and strength fitness has been revealed, for example, the non-linear dose-response relationship between BRI and grip strength. It fills the gap that how fat distribution, rather than total fat, affects adolescent muscle strength. In addition, gender-specific characterization of BRI-strength relationships was showed by using restricted cubic spline models. Distinct patterns in gender was revealed, which addresses a longstanding need to move beyond “one-size-fits-all” obesity and fitness strategies for adolescents. However, there are limitations. Cross-sectional design cannot infer causality between BRI and muscular fitness. Longitudinal study will be designed to develop deeper causality. For instance, a 2-year follow-up to track BRI and strength changes in the same cohort would guide future research and highlight the study's translational potential. Second, self-reported covariate, physical activity (IPAQ-SF) and sleep (PSQI) are subject to recall and reporting bias. Third, sample drawn from three provinces may limit wider applicability. In the future, more provinces and areas will be included in the study. Forth, grip strength were reported as absolute values for the dominant hand only, without adjustment for body mass or inclusion of both hands, which may bias associations. Therefore, in the upcoming investigation and test, we will measure more accurately and collect representative data. Finally, pubertal stage, specific dietary habits (protein intake), and training history or specific exercise types such as resistance training and cardio, were not collected but may influence both BRI and fitness outcomes. While, these factor were not included in the study. Hence, we will consider these information and add them in questionnaires.

## 5 Conclusion

In summary, an appropriate BRI level may contribute to the enhancement of strength-related fitness, while an excessively high BRI could potentially inhibit strength performance. Therefore, health educators should focus on abdominal fat management and promote healthy dietary habits and physical activity. Meanwhile, gender differences provide a basis for personalized intervention strategies, as males and females may require distinct exercise and fat control approaches. This study has several limitations. First, due to the cross-sectional research design, causality could not be established. Future longitudinal studies could better validate the long-term effects of BRI on strength-related fitness. Second, although BRI effectively assesses abdominal fat, factors such as muscle mass, exercise type, and dietary habits may also influence strength performance. Thus, future research should further explore the interactions among these factors. Additionally, the study sample primarily consisted of senior middle school students from three provinces in East China. Future studies could expand the sample scope to include adolescents from diverse regions and cultural backgrounds to enhance the generalizability of the findings.

## Data Availability

The (de-identified) raw data supporting the conclusions of this article will be made available by the authors upon reasonable request, in accordance with applicable ethical and privacy regulations.
